# Potentiation of Calcium Influx and Insulin Secretion in Pancreatic Beta Cell by the Specific TREK-1 Blocker Spadin

**DOI:** 10.1155/2016/3142175

**Published:** 2016-12-25

**Authors:** Céline Hivelin, Sophie Béraud-Dufour, Christelle Devader, Amar Abderrahmani, Sébastien Moreno, Hamid Moha ou Maati, Alaeddine Djillani, Catherine Heurteaux, Marc Borsotto, Jean Mazella, Thierry Coppola

**Affiliations:** ^1^CNRS, Inserm, IPMC, Université Côte d'Azur, Valbonne, France; ^2^CNRS, CHU Lille, Institut Pasteur de Lille, UMR 8199-EGID, Université Lille, 59000 Lille, France; ^3^Département de Physiologie, Institut de Génomique Fonctionnelle (IGF), CNRS/INSERM UMR5203, Université de Montpellier, Montpellier, France

## Abstract

Inhibition of the potassium channels TREK-1 by spadin (SPA) is currently thought to be a promising therapeutic target for the treatment of depression. Since these channels are expressed in pancreatic *β*-cells, we investigated their role in the control of insulin secretion and glucose homeostasis. In this study, we confirmed the expression of TREK-1 channels in the insulin secreting MIN6-B1 *β*-cell line and in mouse islets. We found that their blockade by SPA potentiated insulin secretion induced by potassium chloride dependent membrane depolarization. Inhibition of TREK-1 by SPA induced a decrease of the resting membrane potential (Δ*V*_m_ ~ 12 mV) and increased the cytosolic calcium concentration. In mice, administration of SPA enhanced the plasma insulin level stimulated by glucose, confirming its secretagogue effect observed in vitro. Taken together, this work identifies SPA as a novel potential pharmacological agent able to control insulin secretion and glucose homeostasis.

## 1. Introduction

Insulin secretion by pancreatic *β*-cells is critical for glucose homeostasis. Glucose-induced insulin secretion relies on potassium (K^+^) current-dependent plasma membrane depolarization [1, 2]. Indeed high glucose concentration causes the closure of the ATP-sensitive K^+^ channel (K_ATP_ channel) preventing potassium efflux and leads to *β*-cell membrane depolarization [3–5]. As a consequence, the voltage-dependent calcium channels open, thus allowing calcium influx responsible for the fusion of insulin-containing granules to finally release insulin into the bloodstream [6]. The search for innovative therapeutic strategies improving the *β*-cell function is a key issue for the treatment of diabetes. The first oral antidiabetic agents, metformin and sulfonylureas, were developed in the 1950s and continue to be used effectively. Since 2008, two other very promising therapeutic classes have been placed on the market: dipeptidyl peptidase-4 (DPP-4) inhibitors and glucagon like peptide 1 (GLP-1) analogues to promote insulin secretion without the risk of hypoglycemia. These two drug therapies take advantage of the incretin effect which is a decrease in blood glucose levels. Incretins promote an increase in the amount of insulin released from islets of Langerhans after a meal [7, 8]. Sulfonylureas stimulate insulin secretion by selectively inhibiting *β*-cell K_ATP_ channels [9]. Beside inducing intracellular signaling, GLP-1 also regulates glucose stimulated insulin secretion by the changes in membrane potential [10].

In pancreatic *β*-cells, it is thought that the two-pore potassium channels (K_2_P) participate in regulating cell membrane potential [11–13]. Among K_2_P channels, TREK-1, TREK-2, and TRAAK belong to a subfamily of channels that are opened by mechanical and chemical stimuli [14–16]. TREK-1, the first mechanosensitive K^+^ channel to be identified [17], is activated by polyunsaturated fatty acids and volatile anesthetics [15, 16]. TREK-1 is efficiently inhibited through an intracellular increase of cAMP that leads to the protein kinase A (PKA) activation. Therefore, PKA phosphorylates the serine 333 in the cytoplasmic C-terminal region of the channel [18, 19]. This inhibitory effect is also observed after agonist stimulation of the *β*-adrenergic receptors known to increase intracellular cAMP contents [20]. Incretin hormones like GLP1 and gastric inhibitory polypeptide (GIP) are also able to increase cAMP by activation of PKA [21]. The role of incretins in insulin secretion can therefore be the consequence of both an increase of cAMP and the activation of the phosphorylation of key proteins involved in regulation of insulin exocytosis [22].

Recently, we identified a peptide named spadin (SPA), a partial sequence of the propeptide (PE) released during the maturation of sortilin, as a new class of highly effective and fast acting antidepressants (AD). Sortilin is a class 1 receptor involved in the sorting of several transmembrane proteins including TREK-1 [23]. The AD action of SPA is triggered through the blockade of the TREK-1 channel activity. Since we previously observed that TREK-1 was expressed in the pancreatic *β*-cell line *β*-TC3 [23], we wondered whether TREK-1 and SPA play a physiological role in the regulation of insulin secretion by maintaining the membrane potential of pancreatic cells. In the present work, we demonstrate that SPA by specifically blocking TREK-1 channels depolarizes pancreatic *β*-cell membranes, increases Ca^2+^ influx, and contributes to insulin secretion.

## 2. Materials and Methods

### 2.1. Animals for In Vivo Experiments

All experiments were performed according to policies on the care and use of laboratory animals of European Community Legislation. The local Ethics Committee approved the experiments (protocol number 00893.02).

All efforts were made to minimize animal suffering and reduce the number of animals used. Adult male C57/Bl6 mice, weighing 24–28 g (8–10 weeks old), were used in this study. The animals housed under controlled laboratory conditions with a 12 h dark-light cycle, a temperature of 21 ± 2°C, and a humidity of 60–70% for at least one week prior to drug treatment. Mice had free access to standard rodent diet and tap water.

### 2.2. Cell Culture

Mouse insulin secreting MIN6-B1 cells (passages 35–45) were cultured at 37°C in a humidified atmosphere containing 5% CO_2_ in DMEM medium supplemented with 5% foetal calf serum, 1 mM sodium pyruvate, 2 mM glutamate, 50 mM 2-mercaptoethanol, 100 units/ml penicillin, and 100 mg/ml streptomycin. For Fura-2AM experiments, cells were plated at a density of 1.5 × 10^5^/ml, onto 25 mm poly-D Lysine-coated glass coverslips. For the electrophysiological experiments MIN6-B1 cells were seeded at a density of 20,000 cells/35 mm dishes. Then, cell membrane potential measurements were recorded after 2–6 days of culture.

### 2.3. Whole Cell Patch Clamp Recordings and Membrane Potential Measurements

Whole cell patch clamp recordings were performed in MIN6-B1 cells in a bath solution containing a cocktail of K^+^ channel blockers to record specifically the TREK-1 current. This solution contains 10 mM tetraethyl ammonium (TEA), 3 mM 4-aminopyridine (4-AP), 50 nM charybdotoxin, 10 mM glibenclamide (Glib), and 100 nM apamin.

Membrane potentials were measured in MIN6-B1 cells incubated during 45 min in control conditions in the presence of SPA (1 *μ*M) or Glib (10 *μ*M) or both. Each experimental group was tested in the presence of glucose (2 mM or 10 mM). After the incubation period, cells were patched and membrane potentials were immediately measured using the whole cell patch clamp technique [24]. Each membrane potential was evaluated by using a RK 400 patch clamp amplifier (Axon Instruments, USA), low-pass filtered at 3 kHz and digitized at 10 kHz using a 12-bit analogue-to-digital converter Digidata (1322 series, Axon Instruments, USA). Patch clamp pipettes were pulled using vertical puller (PC-10, Narishige) from borosilicate glass capillaries and had a resistance of 3–5 MΩ. The bath solution contained (in mM) 150 NaCl, 5 KCl, 3 MgCl_2_, 1 CaCl_2_, and 10 HEPES adjusted to pH 7.4 with NaOH. The pipette solution contained (in mM) 155 KCl, 3 MgCl_2_, 5 EGTA, and 10 HEPES adjusted to pH 7.2 with KOH. All experiments were performed at room temperature (21-22°C). Data acquisition was carried out using a microcomputer (Dell Pentium) witch used commercial software and hardware (pClamp 8.2). All values of membrane potentials are expressed in mV as mean ± standard error of the mean (SEM).

### 2.4. Measurement of Cytosolic Calcium Concentrations

The cytosolic calcium variations were measured using the Fura-2AM loading protocol as previously described [25]. Loaded cells were visualized under an inverted epi-fluorescence microscope (AxioObserver, Carl Zeiss, France) using a Fluar 40x/1.3 oil immersion objective. The intracellular Fura-2AM was sequentially excited at 340 and 380 nm with a Xenon lamp through a high-speed multifilter wheel. For each excitation wavelength, the fluorescence emission was discriminated by the same 400 LP dichroic mirror and a 510/40 bandpass filter. Fluorescence images were acquired every 10 sec on an EMCCD camera (Cascade 512, Roper Scientific, Evry, France). Calcium image analyses were made using MetaMorph, MetaFluor (Universal Imaging). Fura-2 fluorescence intensities were expressed as changes relative to the initial fluorescence ratio (F340/380).

### 2.5. Islets Preparation

Mouse islets were isolated by hand-picking after collagenase digestion of pancreas as described previously [26] and were maintained overnight in DMEM supplemented with 10% FCS, 10 mM HEPES, pH 7.4, 1 mM sodium pyruvate, 100 units/ml penicillin-streptomycin, 50 *μ*M *β*-mercaptoethanol, and 11 mM glucose.

### 2.6. Measurement of Insulin Secretion and Cellular Content

For insulin secretion and cellular content, MIN6-B1 cells (5 × 10^5^ per well) or isolated pancreatic islets (20 islets per well) were incubated with 0.1 *μ*M SPA for 45 min at 37°C in control conditions (2.8 mM glucose, 5 mM KCl) or in stimulating conditions (30 mM KCl and 16.7 mM glucose). The amount of insulin was measured using an ELISA kit (Mercodia) as already described [27].

### 2.7. Western Blot Analysis

Solubilized proteins were separated on SDS-PAGE (10% acrylamide) and then transferred to a nitrocellulose membrane that was probed simultaneously with the following primary antibody: a mouse monoclonal sortilin (1 : 1000, BD Transduction Laboratories) and a rabbit polyclonal TREK-1 (1 : 1000, Millipore).

### 2.8. Immunocytochemistry

MIN6-B1 cells were plated on glass coverslips coated with 2 mg/mL poly-L-Lysine. Cells were preincubated for 10 min in Phosphate-Buffered Saline (PBS) and then fixed for 20 min with 4% paraformaldehyde in PBS at room temperature. Coverslips were rinsed twice with PBS and incubated with 50 mM NH_4_Cl in PBS for 10 min to quench excess of free aldehyde groups. After incubation for 20 min in PBS containing 3% Horse Serum (HS) and 0.1% Triton X100, cells were incubated with a rabbit polyclonal anti-TREK1 (1/200, Millipore #AB5860) or a mouse monoclonal anti-chromogranin (1/400, Santa Cruz) for 2 h at room temperature in PBS containing 0.5% HS and 0.1% Triton-X100. Cells were rinsed three times in PBS and incubated for 45 min at room temperature with a Texas-Red-conjugated donkey anti-rabbit antibody (1/400, Invitrogen) or a FITC-conjugated donkey anti-mouse antibody (1/400, Invitrogen) in PBS containing 0.5% HS and 0.1% Triton-X100.

Immunohistochemistry was performed on mouse pancreas slices from wild type and TREK-1 invalidated mice using goat antibodies against insulin (1/400, Santa Cruz Technologies, sc-7838) and rabbit polyclonal anti-TREK1 (1/200, Millipore #AB5860). Briefly, adult male mice were transcardially perfused with 4% paraformaldehyde in PBS and then sacrificed. Pancreas was removed, postfixed in 4% paraformaldehyde in PBS for 2 h at 4°C, and transferred into a 20% sucrose/PBS solution. After freezing of the pancreas in isopentane, 35 *μ*m sections were cut in a cryostat. Sections were stored at −20°C. Labeling was performed as described above for cells.

After two washes with PBS and one with water, coverslips were mounted on glass slides with Mowiol for confocal microscopy examination. Immunofluorescence of confocal images was analysed using ImageJ 1.4.3.67 (WS Rasband, National Institute of Health, https://imagej.nih.gov/ij/).

### 2.9. Intraperitoneal Glucose Tolerance Tests

Intraperitoneal glucose tolerance tests (IPGTTs) were performed on mice after an overnight (16 h) fast. 20 min prior to injection of glucose mice were injected (i.v.) with 100 *μ*l of a saline solution (0.9% NaCl) in the absence or in the presence of 10^−6^ M SPA (8 *μ*g/kg). Glucose administration was performed via intraperitoneal injection (2 g/kg) in 6 to 8 mice for each experimental group. Blood samples (100 *μ*l) were collected from the tail vein before (basal glycemia) and after 10, 20, 30, 60, 90, and 120 min following injection and glucose. Insulin levels were then measured using an ELISA kit as described above.

### 2.10. Statistical Analysis

Data are given as means ± SEM. Statistical significance was evaluated with Student's *t*-test performed using GraphPad Prism.

## 3. Results

### 3.1. SPA Modulates Insulin Secretion in *β*-Cells

**Figure 1:**
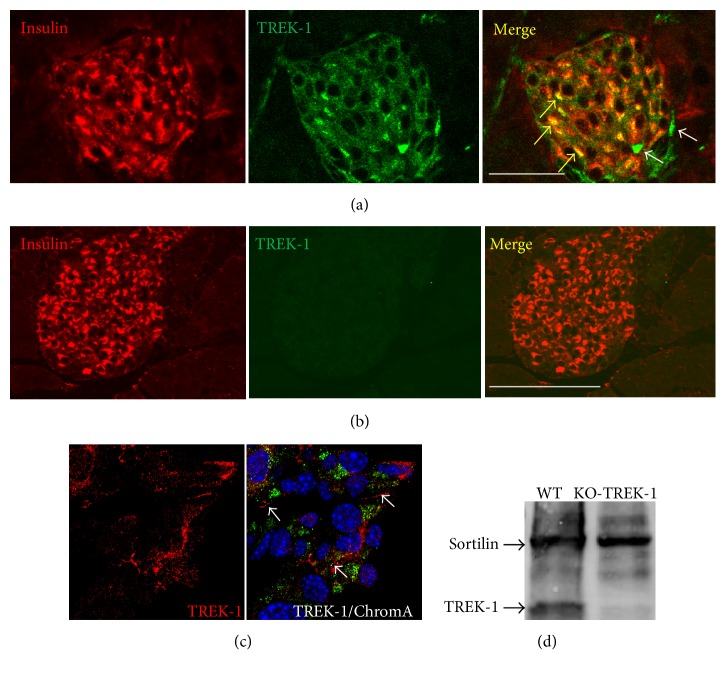
TREK-1 channels are expressed in insulin-containing cells. Immunofluorescent labeling of TREK-1 channels endogenously expressed in mouse pancreatic islets ((a) and (b)) and MIN6-B1 *β*-cells (c). (a) Immunohistochemistry of mouse pancreas sections stained for TREK-1 channels (Alexa-594) and insulin (Alexa-488) as described in Section 2. The merged image indicated colocalization of TREK-1 and insulin (yellow arrows), some peripheral cells were labeled only with TREK-1 antibodies (white arrows). (b) Immunohistochemistry of mouse pancreas sections from TREK-1 KO mice stained for TREK-1 channels (Alexa-594) and insulin (Alexa-488) clearly showed the absence of TREK-1 labeling. (c) Immunocytochemistry of MIN6-B1 cells was performed using anti-chromogranin A (labeling of secretory granules) and anti-TREK-1 antibodies followed by anti-mouse Alexa-488 and anti-rabbit Alexa-594 secondary antibodies and DAPI for nucleus labeling. Merged image showed the expression of TREK-1 at the plasma membrane (white arrows) (scale bar: 100 *μ*m). (d) Immunoblotting of membrane homogenates from islets from WT and KO-TREK-1 mice, with anti-sortilin and anti-TREK-1, reveals a protein of 50 kDa for TREK-1 and 95 kDa for sortilin, respectively.

Previous studies indicated the presence of sortilin in *β*-cell lines and in mouse islets and of TREK-1 in *β*-TC3 cells [23, 28]. We therefore verified, using immunocytochemical and immunohistological approaches, that the channel TREK-1 was also expressed in our models (islets and MIN6-B1 cells).  Figure 1(a) indicated that TREK-1 was expressed in mouse islets visualized by insulin immunoreactivity staining. Control experiments performed on pancreatic slices from TREK-1 KO mice confirmed the specificity of the antibody used (Figure 1(b)).  Figure 1(c) confirmed the expression of TREK-1 in the insulin producing MIN6-B1 cells, particularly at the level of plasma membrane. The specificity of the TREK-1 antibodies used was also confirmed by Western blot analyses of islets proteins from WT and KO-TREK-1 mice (Figure 1(d)).

**Figure 2:**
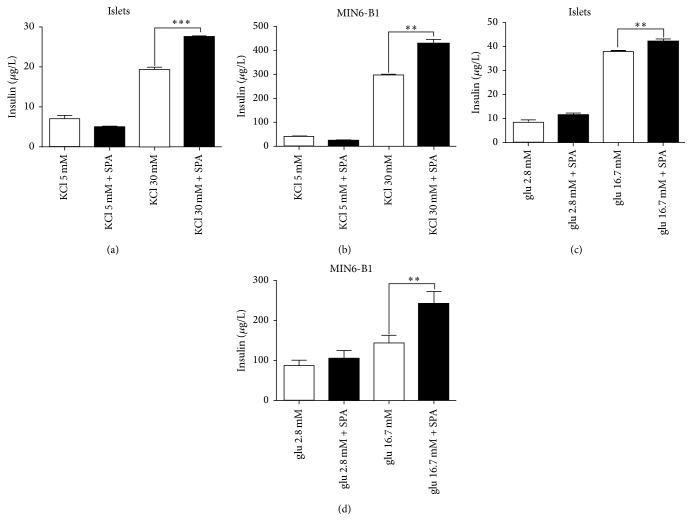
Effects of SPA on insulin secretion from isolated islets and MIN6-B1 cells. Mouse islets (a) or MIN6-B1 cells (b) were incubated at 5 mM (Cont) or stimulating concentration of 30 mM KCl in the presence or in the absence of 10^−7^ M SPA for 45 min. Mouse islets (c) and MIN6-B1 cells (d) were incubated under basal (2.8 mM glucose) or under stimulating (16.7 mM glucose) conditions in the presence or in the absence of 10^−7^ M of SPA for 45 min. The amount of secreted insulin was normalized using the intracellular insulin concentration and was expressed in *μ*g/L. Each value represents the mean ± SEM from 3 independent experiments (^*∗∗*^*p* < 0.01 and ^*∗∗∗*^*p* < 0.001).

The expression of both TREK-1 and sortilin (the precursor of the PE) in *β*-cells and in islets prompted us to investigate the role of the PE related peptide SPA on insulin secretion. Under basal conditions (5 mM KCL, 2.8 mM glucose), incubation of isolated mouse islets and MIN6-B1 cells with 0.1 *μ*M of SPA for 45 min did not modify the amount of secreted insulin (Figures 2(a) and 2(c)). By contrast, SPA potentiated KCl-induced insulin secretion both in islets (from 19.2 ± 0.72 *μ*g/L to 27.47 ± 0.35 *μ*g/L, *p* < 0.001) (Figure 2(a)) and in MIN6-B1 cells (from 297.5 ± 2.92 *μ*g/L to 427.7 ± 20.58 *μ*g/L, *p* < 0.01) (Figure 2(b)). Interestingly, SPA also increased the glucose-induced insulin secretion in mice islets (from 37.8 ± 0.73 *μ*g/L to 47.2 ± 0.9 *μ*g/L, *p* < 0.01) (Figure 2(c)) and in MIN6-B1 cells (from 144.7 ± 18.6 *μ*g/L to 241 ± 30 *μ*g/L, *p* < 0.01) (Figure 2(d)).

### 3.2. SPA Modulates Resting Membrane Potential

**Figure 3:**
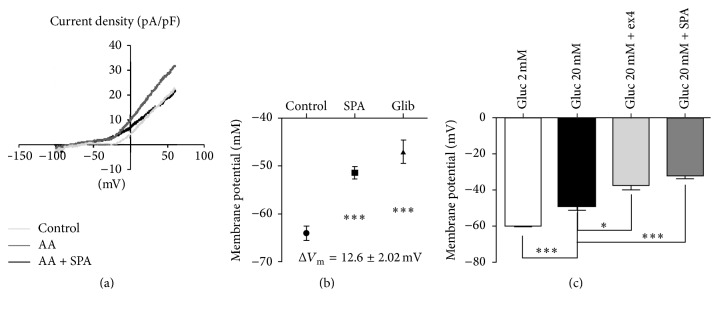
TREK-1 channel current recorded from pancreatic MIN6-B1 cells. (a, b) Whole cell currents measured in MIN6-B1 cells. (a) *IV* curves and membrane potentials recorded after 1 h of incubation in control conditions (grey line), after stimulation of TREK-1 current by 10 *μ*M of arachidonic acid (AA) (dark grey line) and in the presence of 1 *μ*M SPA (dark line). (b) Membrane potential mean values obtained from these conditions; the difference between potential values was Δ*V*_m_ = 12.6 ± 2.02 mV (*n* = 15) between control and SPA (^*∗∗∗*^*p* < 0.001) and Δ*V*_m_ = 17 ± 2.02 mV (*n* = 15) between control and glibenclamide (^*∗∗∗*^*p* < 0.001). (c) Membrane potential mean values obtained from three different groups of cells incubated at low (2 mM) and high (20 mM) glucose concentrations in the absence or in the presence of 1 *μ*M ex4 or 1 *μ*M SPA. ^*∗*^*p* < 0.05 and ^*∗∗∗*^*p* < 0.001.

The resting membrane potential of neuronal cells (i.e., GABA neurons) was known to be maintained in part by TREK-1 channels on which SPA exerted a potent effect [29]. Furthermore, in other cell types such as embryonic atrial myocytes [30] and human osteoblasts [31], TREK-1 contributes to setting the resting membrane potential. Interestingly, it was recently reported that two members of the K_2_P family (TALK-1 and TASK-1) are expressed in pancreatic islets [32, 33] in which they modulate electrical activity. We therefore postulated that these background K^+^ channels could function as modulators of *β*-cell excitability. To answer this question we tested the effects of SPA on K^+^ current recorded on whole MIN6-B1 cell patch. As shown in Figure 3(a), TREK-1 current was potentiated by 10 *μ*M arachidonic acid (AA) and application of SPA (10^−6^ M) inhibited the AA activated TREK-1 current. This SPA effect was summarized in Figure 3(b) where the difference of membrane potential of SPA-treated versus control cells (*E*_mSPA_ − *E*_mC_) Δ*E*_m_ was 12.6 ± 2.0 mV (*n* = 15, *p* < 0.001). As a control we observed that glibenclamide induced a depolarization up to −47.07 ± 2.33 mV (*p* < 0.001) (Δ*E*_m_ = 17 ± 2.3 mV, *p* < 0.001) (Figure 3(b)). We have also tested the SPA effect when added to glucose 20 mM. As expected, we observed a strong and robust depolarization when the MIN6-B1 cells were incubated in the presence of 20 mM glucose, −60 ± 1.0 mV (2 mM glucose) versus −48.71 ± 2.378 mV (20 mM glucose) (Figure 3(c)). Addition of the GLP-1R agonist exendin4 (ex4) (10^−7^ M) induced a significant additive effect to reach −37.60 ± 2.50 mV (*p* < 0.05). Interestingly, SPA effects were also additive to reach −32 ± 1.826 mV (*p* < 0.001) when coincubated with 20 mM glucose (Figure 3(c)).

### 3.3. Effect of SPA on Intracellular Calcium Content

**Figure 4:**
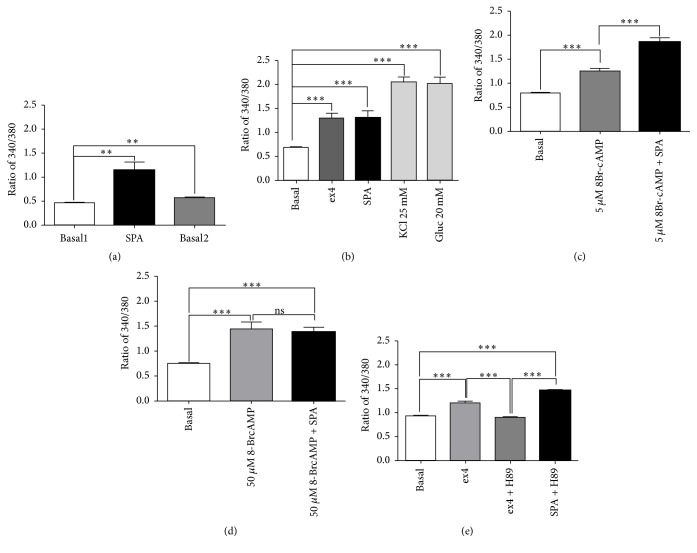
Effects of SPA on cytosolic calcium concentrations. Cytosolic calcium variations were measured using the Fura-2AM in the mouse MIN6-B1 *β*-cell line. Fura2-AM absorbance ratio (340/380) was given for the time point with the maximal signal. (a) At low glucose concentration (5 mM), SPA (10^−7^ M) induced a significant cytosolic calcium rise (*n* = 18, *p* < 0.01) that did not return to basal level after washing out (*p* < 0.01). (b) Comparing SPA and ex4 effects, controls were performed using either KCl (25 mM) or glucose (20 mM) (*n* = 17). (c) Preincubation of MIN6-B1 cells with 5 mM 8Br-cAMP did not prevent the stimulating effect of SPA on calcium rise (*n* = 48). (d) When MIN6-B1 cells were preincubated with 50 mM 8Br-cAMP, SPA was not able to increase intracellular calcium (*n* = 12). (e) Preincubation of MIN6-B1 cells in the presence of 1 *μ*M H89 significantly inhibited the ex4 effect but not that of SPA (*n* = 38). *n* indicates the number of responding cells in each of three experiments. Results are expressed as mean ± SEM; ^*∗∗*^*p* < 0.01, ^*∗∗∗*^*p* < 0.001, and ns: nonsignificant.

After recording of cultured MIN6-B1 *β*-cells, we observed that, as in INS1E *β*-cells [25], SPA (10^−7^ M) induced an increase in intracellular calcium level (ratio 340/380 value: 1.15 ± 0.16), which did not return at the basal level after withdrawal (value: 0.46 ± 0.01 versus 0.69 ± 0.032, before and after SPA, resp.) (Figure 4(a)). Since SPA increased the glucose-induced insulin release like incretins, we compared the effect of ex4 with that of SPA. Ex4 induced an increase of intracellular calcium level (value: 1.29 ± 0.1, *p* < 0.001) as well as SPA (1.30 ± 0.14, *p* < 0.001) when compared with the basal level (value: 0.61 ± 0.02). As controls, we measured the effect of KCl (25 mM) and glucose (20 mM) on intracellular calcium levels and obtained ratio values of 2.05 ± 0.11 (*p* < 0.001) and 2.0 ± 0.11 (*p* < 0.001), respectively (Figure 4(b)). To verify the involvement of intracellular cAMP on the SPA effect, we used various concentrations of the stable analogue 8-Br-cAMP. At low dose (5 *μ*M) the cAMP analogue increased calcium levels from a ratio of 0.8 ± 0.03 to 1.25 ± 0.06 (*p* < 0.001) (Figure 4(c)). However, addition of SPA (10^−7^ M) enhanced the signal values up to 1.87 ± 0.07 (*p* < 0.001 versus 5 *μ*M 8Br-cAMP alone) (Figure 4(c)). A higher dose of 8-Br-cAMP (50 *μ*M) induced an increase of cytosolic calcium (from 0.75 ± 0.02 to 1.44 ± 0.14, *p* < 0.001) that was not significantly modified by addition of SPA (ratio of 1.39 ± 0.1) (Figure 4(d)). Interestingly, the PKA inhibitor H89 inhibited the ex4 effect but not the SPA effect on calcium levels (Figure 4(e)). This indicates that the action of SPA on intracellular calcium levels is not dependent on PKA activity.

### 3.4. SPA Improves Plasma Insulin Level and Leads to Hypoglycemia in Mice

**Figure 5:**
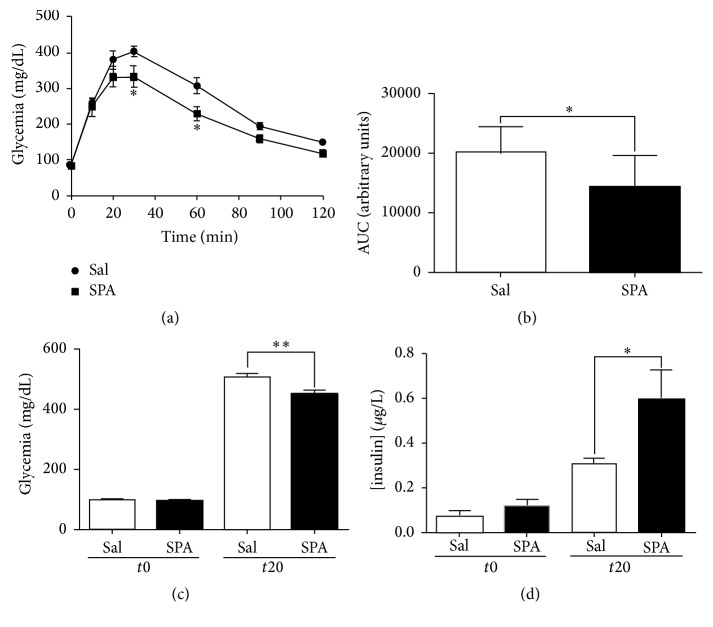
SPA modulates insulin secretion in mice. (a) IPGTT challenge (2 g/kg glucose i.p.) is performed onto two groups of C57Bl6 mice. 20 minutes before glucose injection, mice were injected (i.v.) with SPA (8 *μ*g/kg) (square dots) or saline (round dots). Glycemia was measured at time 0 (before injection) and 10, 20, 30, 60, 90, and 120 minutes after glucose injection, from blood samples collected from the caudal vein tail (*n* = 9 for saline and *n* = 7 for SPA). (b) The areas under curve (AUC) were calculated using GraphPad Prism from the mean of individual AUC obtained for each mouse. (c) Glycemia was measured from blood samples collected before i.v. injection of SPA (*n* = 12) or saline (*n* = 13) (*t*0) and 20 minutes after i.p. glucose injection (*t*20). (d) Plasma insulin concentration was measured from the same blood samples as above (^*∗∗*^*p* < 0.01, ^*∗*^*p* < 0.05).

To investigate the role of SPA on glycemia in mice, we challenged the action of i.v. injection of SPA (100 *μ*L of 1 *μ*M, 8 *μ*g/kg) during the glucose tolerance test. We followed the glucose serum concentration up to 120 min after i.p. injection of a high glucose solution (2 g/kg). In control conditions (injection of 100 *μ*L saline), we observed a typical response with a blood glucose concentration that increased from the injection time up to 20–30 min after injection followed by the return to the basal level after 120 min (Figure 5(a)). In mice injected with SPA 20 min before the test, the increase in blood glucose concentration was smaller to reach a maximal concentration of 332.6 ± 31.26 mg/dL (*n* = 7) compared to 404.1 ± 16.32 mg/dL (*n* = 9) in the control condition (*p* < 0.05) (Figure 5(a)) at 30 min. At 60 min, the glycemia remained statistically lower in SPA-injected mice (314.8 ± 26.04 mg/dL, *n* = 9 versus 229.3 ± 20.55 mg/dL, *n* = 7, *p* < 0.05) (Figure 5(a)). These differences were illustrated by the smaller area under the curve (AUC) decreased by 28.46% in the presence of SPA (20205 ± 1449 arbitrary unit (AU), *n* = 9 for control versus 14455 ± 2015 AU, *n* = 7 for SPA) (*p* < 0.05) (Figure 5(b)). In order to investigate the correlation between the SPA-induced effect on glycemia and the amount of insulin released in the blood, we measured both glucose and insulin from blood samples collected before and 20 min after glucose injection. We confirmed that SPA significantly decreased glycemia 20 min after glucose injection (503 ± 12.9 mg/dL (*n* = 13) for saline versus 450.4 ± 10.15 mg/dL (*n* = 13) for SPA) (*p* < 0.005) (Figure 5(c)). In parallel, SPA significantly increased glucose-induced serum insulin content (from 0.31 ± 0.03 *μ*g/L (*n* = 13) to 0.59 ± 0.13 *μ*g/L (*n* = 12) (*p* = 0.029) (Figure 5(d)).

## 4. Discussion

The present work describes the effect of SPA in the regulation of both glucose homeostasis and glucose-induced insulin secretion. Although SPA displays an effect similar to those of incretins, its mechanism of action is quite different since it is not controlled by the intracellular levels of cAMP.

Developing safer drugs and drug candidates for the treatment of diabetes is one of the major goals of the pharmaceutical industry, and its importance has been intensified by the withdrawal of many drugs from the market due to side effects and a decrease of new chemical entities introduced into the market [34]. We initially developed SPA as a TREK-1 channel blocker that could be used for the treatment against mood disorders. In that perspective, we have established that SPA, designed from an endogenous peptide, is a safer choice for a new pharmacological concept due to its absence of toxicity and long lasting central side effects [35, 36]. Since TREK-1 is expressed in *β*-cells, we investigated the role of SPA in the glucose homeostasis to verify that this compound does not display any deleterious peripheral action [35]. In the present work, we showed that SPA could have further interesting functions in the regulation of glucose homeostasis.

Endocrine pancreatic islets can be compared in some instance to neurons. Indeed, like neurons, *β*-cells secrete insulin after depolarization of plasma membrane upon stimulation. The secretion process is controlled by ATP-dependent potassium channels that are already a target in the development of type 2 antidiabetic drugs. The background two-pore potassium channel TREK-1 could also be an actor in the regulation of membrane potential. In neurons, TREK-1 channels are important regulators in the signaling of serotonin (5-HT) or gamma amino butyric acid (GABA), two neurotransmitters involved in brain pathophysiology [37]. Therefore, SPA, as a TREK-1 blocker, is able to improve brain functions [29]. In the present work, we demonstrated that TREK-1 channels are expressed in endocrine pancreas (Figure 1) and that SPA enhances stimulated insulin secretion both in islets and in MIN6-B1 cells (Figure 2). Deciphering the effect of SPA on *β*-cells by using the MIN6-B1 cell line, we observed that the peptide is able to induce the depolarization of the plasma membrane with the same potency than glibenclamide (a K_ATP_ channel blocker) and ex4 (a GLP1 receptor agonist) (Figure 3). This plasma membrane depolarization likely facilitates the exocytosis process through the enhancement of intracellular calcium concentration (Figure 4). SPA does not induce insulin secretion at low glucose concentration, because it does not reach the threshold of depolarization necessary to induce exocytosis. *β*-cell does not induce an action potential even though more than 90% of the K_ATP_ channels are closed [6]. We hypothesize that TREK-1 depolarization is not enough to induce a robust calcium entry. As insulin secretion is a calcium dependent process, exocytosis needs a stronger increase in calcium concentrations to trigger fusion of insulin granules membrane with the plasma membrane. Since SPA increases the glucose-induced insulin secretion, we hypothesized that this peptide could function like an incretin hormone. However, as expected, H89 inhibits the effects of ex4 on calcium influx but not those of SPA, indicating that its action is not mediated by PKA and likely results only from the blocking of K^+^ currents. We propose that TREK-1 currents can be blocked by SPA or by PKA dependent phosphorylation. In this way, we can hypothesize a direct blocking effect of SPA on TREK-1 activity whereas GLP1 agonists blocking action on these channels are the consequence of their effects on PKA activation.

Finally, in vivo, we clearly observe an action of SPA on glycemia since the glucose level is always lower in mice treated with the peptide during IPGTT experiments. During the glucose tolerance tests, SPA significantly increases plasma insulin concentration indicating that the lower level of glycemia is likely the consequence of insulin amount.

In conclusion, this work constitutes the first report on the involvement of TREK-1 channels in the function of *β*-cells particularly the secretion of insulin. Interestingly, the activity of these channels can be modulated by SPA leading to an incretin like action independent from the activation of PKA. Therefore, this peptide could be the basis for the development of new therapeutic strategies for the treatment of diabetes.
